# Aneuploidy in yeast: Segregation error or adaptation mechanism?

**DOI:** 10.1002/yea.3427

**Published:** 2019-08-01

**Authors:** Ciaran Gilchrist, Rike Stelkens

**Affiliations:** ^1^ Division of Population Genetics, Department of Zoology Stockholm University Stockholm Sweden

**Keywords:** adaptation, aneuploidy, environmental stress, hybridisation, yeast

## Abstract

Aneuploidy is the loss or gain of chromosomes within a genome. It is often detrimental and has been associated with cell death and genetic disorders. However, aneuploidy can also be beneficial and provide a quick solution through changes in gene dosage when cells face environmental stress. Here, we review the prevalence of aneuploidy in *Saccharomyces*, *Candida*, and *Cryptococcus* yeasts (and their hybrid offspring) and analyse associations with chromosome size and specific stressors. We discuss how aneuploidy, a segregation error, may in fact provide a natural route for the diversification of microbes and enable important evolutionary innovations given the right ecological circumstances, such as the colonisation of new environments or the transition from commensal to pathogenic lifestyle. We also draw attention to a largely unstudied cross link between hybridisation and aneuploidy. Hybrid meiosis, involving two divergent genomes, can lead to drastically increased rates of aneuploidy in the offspring due to antirecombination and chromosomal missegregation. Because hybridisation and aneuploidy have both been shown to increase with environmental stress, we believe it important and timely to start exploring the evolutionary significance of their co‐occurrence.

## 1 BACKGROUND

During cell division, cells can gain or lose a chromosome, resulting in abnormal chromosome numbers that do not match their parental karyotypes. This is known as aneuploidy. Aneuploidy is a common by‐product of chromosomal missegregation during meiosis and mitosis. It has differential effects on cell fitness, arguably most of them detrimental. In humans, aneuploidy has been linked to genetic disorders including Down's syndrome (Dunlap, Aziz, & Rosenbaum, 1986; Hassold & Hunt, 2001), cancer (Gao et al., 2007; Gao et al., 2016; Kops, Weaver, & Cleveland, 2005), and various forms of karyotype mosaicism (Biesecker & Spinner, 2013). In Saccharomyces cerevisiae, aneuploidy of the largest chromosome (Chr IV) can increase cell doubling time by 167%, slowing down growth dramatically (due to a delay at the G1 stage of mitosis caused by the larger DNA content; Torres et al., 2007). At the same time, aneuploidies of other chromosomes can lead to enhanced proliferative capacity in *Saccharomyces*, conferring faster growth compared with the corresponding euploid strains under specific conditions (Zhu, Pavelka, Bradford, Rancati, & Li, 2012). Similarly, aneuploid cancer cells often have competitive advantages over euploid cells (Sheltzer & Amon, 2011) and increased metastatic success, spreading from one type of tissue to another (Gao et al., 2016).

Recently, a growing number of studies link aneuploidy to adaptation, suggesting that aneuploidy (as a result of nondisjunction during mitotic division) may provide a natural, but transient, route to rapid adaptive evolution in microbial populations (Chang, Lai, Tung, & Leu, 2013; Hose et al., 2015; Smukowski Heil et al., 2017). For instance, several experimental evolution studies using yeast (Chen, Bradford, Seidel, & Li, 2012; Gorter et al., 2017; Pavelka et al., 2010; Selmecki et al., 2015; Selmecki, Dulmage, Cowen, Anderson, & Berman, 2009; Yona et al., 2012) and other pathogenic fungi such as Candida albicans (Selmecki, Forche, & Berman, 2006) and Cryptococcus neoformans (Gerstein et al., 2015) suggest that aneuploidy can confer increased stress and drug resistance. This is likely due to the variation in gene dosage caused by the loss or gain of chromosomes causing higher phenotypic diversity or pathogenic potential in aneuploid microbial populations (Bader et al., 2012; Beach et al., 2017; Gerstein et al., 2015; Hirakawa, Chyou, Huang, Slan, & Bennett, 2017; Hu et al., 2011; Ni et al., 2013).

**Figure 1:**
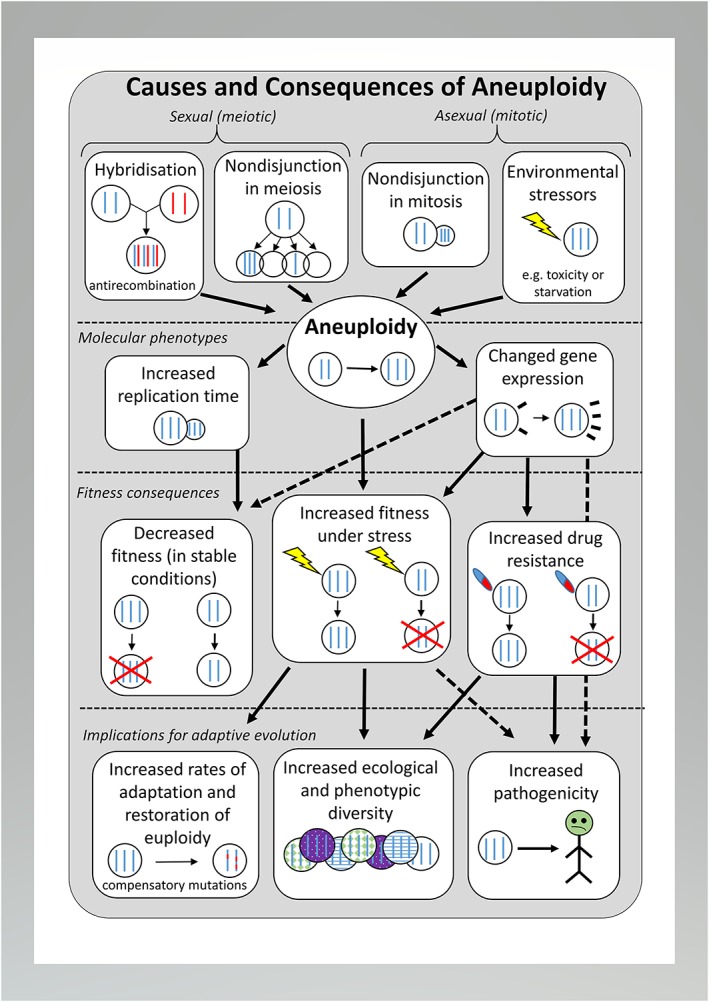
Diagram of the causes and consequences of aneuploidy. Sexual reproduction promoting aneuploidy includes hybridisation between divergent species followed by antirecombination during hybrid meiosis, and nondisjunction of sister chromatids in regular nonhybrid meiosis, leading to chromosomal missegregation. Aneuploidy can also occur due to nondisjunction during mitotic cell division and when strains are propagating under severe environmental stress. Solid arrows show known causal relationships; dashed arrows show hypothesized relationships

In this review, we analyse patterns and frequencies of chromosome aneuploidy in experimental, industrial, and pathogenic yeast strains. We also analyse relationships between aneuploidy, chromosome size, and specific environmental stressors (Figure 1) and discuss whether aneuploidy may provide populations with the phenotypic variation needed to adapt to stressful or quickly changing environments.

In addition, we would like to highlight an interesting but unstudied research question. We think it is important and timely that we start exploring the connection between hybridisation, aneuploidy, and adaptation in microbes. Chromosomal nondisjunction is known to be a particularly prevalent outcome of interspecific hybrid meiosis in yeast, leaving a large fraction of the F1 hybrids offspring (spores) aneuploid (Boynton, Janzen, & Greig, 2018; Greig, Travisano, Louis, & Borts, 2003; Rogers, McConnell, Ono, & Greig, 2018). Aneuploidy is thus thought to be one of the main reasons for F1 hybrid sterility in yeast, causing almost complete reproductive isolation between species of the *Saccharomyces* sensu stricto complex (F1 hybrids produce less than 1% viable gametes (Hou, Friedrich, de Montigny, & Schacherer, 2014; Liti, Barton, & Louis, 2006). Although these F1 hybrids can persist mitotically for hundreds to thousands of generations, F1 hybrid meioses can produce high fitness aneuploid offspring with enhanced adaptive potential (e.g., in interspecific *Crypococcus* hybrids (Hu et al., 2011; Lengeler, Cox, & Heitman, 2001). Although little data exist to date, we explore the idea that some aneuploid hybrids may be evolutionary successful and could outcompete euploid, nonhybrid strains, especially in highly stressful environments. This has particular relevance for hybrid pathogenic microbes and traits related to their epidemiology.

## 2 ANEUPLOIDY FACILITATES ADAPTATION

In this section, we provide a summary of what is known about the adaptive potential of aneuploidy through mitotic nondisjunction during asexual reproduction in yeast. We include examples from laboratory, industrial, and pathogenic strains adapting to different kinds of stress and focus on complete aneuploidy where an entire chromosome is gained or lost from the cell. Some studies reviewed here also include examples where large parts of a chromosome are amplified or deleted, which is referred to as partial aneuploidy (Selmecki et al., 2006; Sunshine et al., 2015). We will specifically mention if results are based on partial aneuploidies.

### 2.1 Aneuploidy in laboratory strains

**Figure 2:**
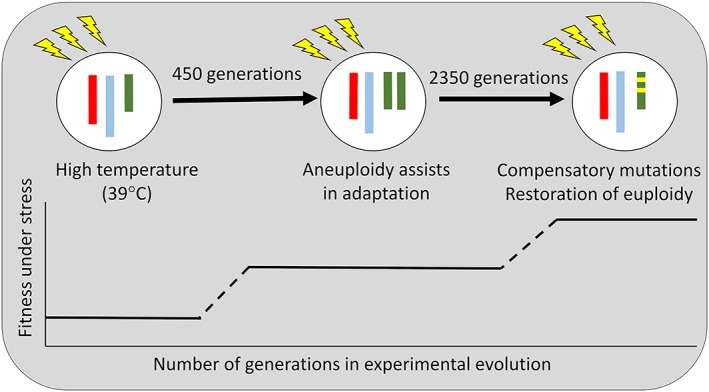
Aneuploidy as a temporary adaptation mechanism Redrawn from (Yona et al., 2012). When repeatedly subjected to high temperatures (yellow bolts), replicate diploid yeast populations (circles) gained an extra chromosome (red, blue, and green lines; only three of 16 chromosomes shown) within 450 generations of long‐term exposure to high temperatures. After 2,350 generations of exposure to high temperatures, euploidy was restored and novel beneficial mutations (indicated in yellow) compensated for the loss of the extra chromosome and increased fitness further

Since before the advent of genomics, a role for aneuploidy in adaptation in yeast has been suggested (Cox & Bevan, 1962). The consistency with which aneuploid cells are recovered following the exposure of yeasts to stress in the laboratory suggests that it can serve as an adaptation mechanism to harsh environments (see Box [Link] and Table 1 for more detailed information). In S. cerevisiae, adaptation though aneuploidy is likely due to the amplified or modified expression of genes on aneuploid chromosomes, which can lead to increased tolerance to environmental stress (Chen et al., 2012; Lauer et al., 2018; Linder, Greco, Seidl, Matsui, & Ehrenreich, 2017; Millet, Ausiannikava, Le Bihan, Granneman, & Makovets, 2015; Selmecki et al., 2015; Yona et al., 2012). This includes low nutrient availability (Gresham et al., 2008; Hong & Gresham, 2014; Selmecki et al., 2015; Sunshine et al., 2015), high ethanol concentrations (Voordeckers et al., 2015), high temperature (Figure 2 adapted from Yona et al., 2012), and telomerase insufficiency (Millet et al., 2015). Box [Link] provides a more detailed overview of common aneuploidies in *Saccharomyces* and *Candida* and what is known about their respective adaptive potential.

**Table 1:** Common aneuploidies linked with adaptation to different types of stress in Saccharomyces cerevisiae

Chromosome	Type of aneuploidy	Stress	Selection agent	Ploidy	Notes	Source
II, III, and V	+	Environmental stress	Paraquat (superoxide)	1N		Jonas Warringer, pers. Comm.
III	+	Environmental stress	Ethanol	2N	Near lethal stress	Voordeckers et al. (2015)
III	+	Temperature	Heat (39°C)	1N	Transient (eliminated within 2,350 gens)	Yona et al. (2012)
IV	+	Environmental stress	Hydrogen Peroxide (oxidative)	1N		Linder et al. (2017)
V	+	Environmental stress	High pH (8.6)	1N	Transient (eliminated within 2,350 gens)	Yona et al. (2012)
VIII	−	DNA damage	Telomerase Insufficiency	2N	Allows for survival	Millet et al. (2015)
VIII	+	Drug resistance	Fluconazole (antifungal)	2N		Chen et al. (2012)
IX	+	Drug resistance	Hygromycin B	2N		Chen et al. (2015)
XII	−	Drug resistance	Benomyl	2N		Chen et al. (2012)
XIII	+	Starvation	Raffinose	4N		Selmecki et al. (2015)
XIII	+	Toxic metabolic intermediates	Galactose	1N and 2N	Recovery after knockout of GAL7	Sirr et al. (2015)
XIV	+	Starvation	Allantoin	2N		Hong and Gresham (2014)
XV	+	Proteotoxic stress	Radicol (Hsp90 inhibitor)	1N and 2N		Chen et al. (2012)
XVI	−	Drug resistance	Tunicamycin	2N		Chen et al. (2012)

*Note*. Review of published data showing chromosome numeral, type of aneuploidy (amplification or deletion is + or −, respectively), type of stress, selection agent, initial ploidy of the experimental population, notes, and references.

Box 1 **Aneuploidy in response to different types of stress**

**Temperature**

When exposed to high temperatures (39°C), three replicate S. cerevisiae populations showed aneuploidy of Chr III after 450 generations (Yona et al., 2012). However, by 1,000 generations, all populations lost their extra copy of Chr III, instead retaining increased expression of 17 genes within a region of Chr III (Figure 2). The expression of 13 of these genes clearly assisted in heat tolerance when upregulated. The same pattern of aneuploidy followed by reversal to euploidy occurred when exposing populations to high pH, suggesting that aneuploidy may be a “quick fix” allowing survival with a temporarily more costly strategy in the short term. Consistent with this, the same study also found that if stress was applied more gradually, for example, when increasing temperature in small increments, aneuploidy did not occur at all (Yona et al., 2012).

**Starvation**

Aneuploidy, both partial and complete, can amplify the expression of genes involved in general adaptation to starvation in S. cerevisiae populations grown under sulphate‐, glucose‐, phosphate‐, carbon‐, and nitrogen‐limited conditions (Gresham et al., 2008; Hong & Gresham, 2014; Selmecki et al., 2015; Sunshine et al., 2015). In extreme cases, every population grown in sulphate‐limited conditions contained partial or complete aneuploidy (Sunshine et al., 2015).

**Toxic stress**

Chr III has been repeatedly found to be both completely and partially aneuploid in S. cerevisiae populations adapted to high levels of ethanol (Morard et al., 2019; Voordeckers et al., 2015). Industrial *Saccharomyces* strains, especially those used for brewing and distilling, frequently experience aneuploidisation, likely for the same reason—the toxicity of high alcohol concentrations in the fermentation vessel (Gorter de Vries, Pronk, & Daran, 2017). Aneuploidy also confers stress tolerance in the spoilage yeast *Zygosaccharomyces rouxii*, perhaps allowing them to thrive in high salt and sugar environments (Solieri, Dakal, & Bicciato, 2014). Tolerance to hydrogen peroxide has been linked with Chr IV aneuploidy in S. cerevisiae. This was linked with increased copy number of the gene TSA2, which is located on Chr IV (Linder et al., 2017). Although S. cerevisiae is normally able to utilise galactose as a food source, strains with the GAL7 gene knocked out are highly sensitive to galactose. In rare cases, these knockout strains may evolve resistance. An analysis of 247 genomes of these haploid galactose‐resistant strains showed that 124 (~50%) were aneuploid, with an extra Chr XIII in 93 strains (75% of the aneuploid strains; Sirr et al., 2015). A gene on Chr XIII (GAL80) was linked with this resistance.

**Telomerase insufficiency**

Telomerase is a reverse transcriptase enzyme that adds repetitive sequences to the 3′ end of telomeres, protecting chromosomes from DNA damage. When cell proliferation in S. cerevisiae is limited by low amounts of telomerase in cells grown at elevated temperature, 32 of 79 surviving clones had changed from haploid to near diploid karyotypes after 80 generations (Millet et al., 2015). The near diploid clones had only a single copy of Chr VIII and produced colonies smaller than the wild type (Millet et al., 2015). However, often these strains produced wild‐type‐like colonies again, which contained two copies of all chromosomes. This suggests they had exploited the adaptive advantage of their aneuploidy to survive the period of telomerase insufficiency and then became fully diploid again. The authors suggested that aneuploidy of Chr VIII affected the genes PRP8, UTP9, KOG1, and SCH9, whose loss results in a reduction in expression of ribosomal components to reduce their overall proportion, which may assist in the adaptation to reduced telomerase expression.

Box 2 **Common aneuploidies**

In S. cerevisiae, aneuploidy can lead to the formation of multicellular structures that produce “fluffy” colonies on agar plates (Tan et al., 2013). This included four chromosomes, Chr III, Chr X, Chr XV, and Chr XVI, with an intermediate phenotype produced by aneuploidy of Chr V. This aneuploidy‐dependent phenotype has also been found in C. albicans, where an extra copy of Chr IV is responsible for filamentation during growth, which also increased the virulence of the strain (Hirakawa et al., 2017).

Whole genome sequencing of 1,011 S. cerevisiae isolates from around the world, including wild, domestic, and clinical strains, showed that 19.1% of the isolates contained aneuploidies for one or more chromosomes (Peter et al., 2018). Interestingly, this study found the same four chromosomes to be frequently aneuploid as a previous study on pathogenic S. cerevisiae strains (Zhu, Sherlock, & Petrov, 2016). Zhu et al. (2016) found Chr XI (667 kb) to be most often aneuploid, followed by Chr I (231 kb), Chr VIII (563 kb), and Chr IX (440 kb). In addition, Peter et al. found Chr III, which is one of the smallest chromosomes (317 kb), to often be aneuploid, whereas Chr IV, the largest (1532 kb), was aneuploid the least often. The large Chr IV was also less frequently aneuploid in viable F1 S. cerevisiae × *Saccharomyces* paradoxus hybrid spores compared with other chromosomes (Kao, Schwartz, & Sherlock, 2010). Our survey of eight articles also found Chr IV to be aneuploid the least common (Table S1). Chr IV has previously been associated with a 167% increase in doubling time when it is aneuploid under normal growth conditions, the largest increase for any chromosome (Torres et al., 2007). This suggests that the fitness cost of aneuploidy for Chr IV leads to its low frequency under most conditions, though it has been found to assist in adaptation on occasion (Linder et al., 2017).

After continued growth and exposure of S. cerevisiae to the drug radicicol, which inhibits the chaperone complex Hsp90, three resistant diploid populations emerged, through gaining of an extra copy of Chr XV (Chen et al., 2012). Haploid strains containing two copies of Chr XV were also resistant to radicicol. Hsp90 stress generates more chromosomal instabilities, resulting in other aneuploidies in addition to that of Chr XV. Pretreatment with radicicol increased growth in fluconazole, tunicamycin, and benomyl (Chen et al., 2012). Interestingly, Chr XV aneuploidy has been shown to also cause sensitivity to the drug hygromycin B (Chen et al., 2015). When subjected to hygromycin B, aneuploid strains shed their extra copy of Chr XV whereas others gained an extra copy of Chr IX, resulting in populations with a mixture of euploid and aneuploid cells. This shows that the fitness benefits that aneuploidy can potentially provide are extremely dependent on the environment in which the strains grow.

### 2.2 Aneuploidy in industrial strains

Aneuploidy is common in *Saccharomyces* strains used for industrial purposes, such as fermentation and baking. In total, there were 604 cases of aneuploidies in the 287 aneuploid industrial strains we reviewed here (Duan et al., 2018; Gallone et al., 2016; Peter et al., 2018; Table S2A). Whether these aneuploidies are due to adaptation to industrial conditions or by‐products of selection on other traits (e.g., flavour of fermentation product) is not known.

One commonly studied species is *Saccharomyces pastorianus*, which was originally the result of hybridisation between S. cerevisiae and *Saccharomyces eubayanus* (Peris et al., 2016) and is used in the production of all lager beers. The *S. pastorianus* genome is well known to contain various aneuploidies. Overall, chromosome copy number varies drastically from 45 to 79 between *S. pastorianus* strains, that is, from near triploid (3N) to almost pentaploid (5N; van den Broek et al., 2015). This variation may lead to differing properties and flavours made during fermentation. In addition, an investigation of partial aneuploidies in *S. pastorianus* found examples, which included genes linked with flocculation (Dunn & Sherlock, 2008). This is not surprising, as flocculation is an important part of the brewing process in lager.

Aneuploidy has also been found in the yeasts used for the distilling of spirits such and vodka and whisky (Adamczyk et al., 2016; Deregowska et al., 2015; Naumova, Sadykova, Martynenko, & Naumov, 2013). Aneuploidies in distilling strains have been linked with increased copy number of MAL and SUC genes, which are known to play important roles in the distilling process (Naumova et al., 2013).

### 2.3 Aneuploidy in pathogenic yeasts

During infection, pathogens are confronted with harsh new environments inside their hosts. The host's attempts at defence include phagocytosis (digestion of cells by phagocytes) and the adaptive immune response, resulting in a stressful situation for the invading pathogen (Romani, 2004). In response, some pathogenic cells change ploidy or become aneuploid upon invasion of their host (reviewed in Todd, Forcher, & Selmecki, 2017).

#### 2.3.1 Saccharomyces

Although S. cerevisiae is generally not harmful to humans, it can become an opportunistic pathogen in immunocompromised individuals. A study of 136 such strains found that 36% contained aneuploidies (Zhu et al., 2016). Our analysis of published data from Zhu et al. (2016) and Peter et al. (2018) found a total of 146 aneuploidies in 73 pathogenic aneuploid S. cerevisiae strains (Table S2B).

#### 2.3.2 Candida

C. albicans is the most common fungal pathogen in humans, causing severe illness in immunocompromised patients, mostly through infection of the mucous membranes. C. albicans can undergo a parasexual cycle, during which two diploid cells form a tetraploid cell. These cells either reproduce asexually as tetraploids or revert to diploid or near diploid haplotype, through a concentrated chromosome loss event (Bennett & Johnson, 2003). This parasexual cycle frequently generates a vast variety of aneuploid offspring (Hickman, Paulson, Dudley, & Berman, 2015) with more variation in phenotype than euploid strains (Hirakawa et al., 2017). Although these aneuploidies were detrimental under normal conditions, in some cases, they assisted in increasing virulence and resistance to fluconazole, with an extra Chr III or Chr VI being most common (Forche, Magee, Selmecki, Berman, & May, 2009).

In C. albicans strains that are resistant to the antifungal drug fluconazole, a specific aneuploidy is often recovered, which is the formation of an isochromosome containing two copies of the left arm of Chr V (i5L; Selmecki et al., 2006). The increased copy number of two genes (ERG11 and TAC1) located in this region has been shown to assist with the fluconazole adaptation in an elegant follow‐up experiment (Selmecki, Gerami‐Nejad, Paulson, Forche, & Berman, 2008). Interestingly, showcasing the paradoxical fitness effects of aneuploidy, i5L reduces resistance to another type of drug (pyrvinium pamoate) in C. albicans, (Chen et al., 2015). In strains of C. glabrata, a pathogen of the bloodstream and the urogenital tract (Ahmad et al., 2013; Poláková et al., 2009), aneuploidy (including an entirely new chromosome) has also been found to confer resistance to fluconazole and was also suggested to assist in dealing with stressful host defence mechanisms during infection.

#### 2.3.3 Cryptococcus

*Cryptococcus* species are the causative agents of cryptococcal meningitis and a significant source of mortality. In a study investigating 188 mostly clinical isolates of C. neoformans var. *grubii*, 25 of 164 haploid isolates showed partial or complete aneuploidy (Rhodes et al., 2017). Variation in chromosome copy number in C. neoformans has been linked to an increase of virulence in mice and HIV/AIDS patients (Hu et al., 2011). In C. neoformans, cells of the same mating type are able to cross, resulting in diploid cells. When these diploids sporulate, this sometimes results in haploid or aneuploid offspring, which contain large amounts of phenotypic variability, for example, showing increased melanin production and increased fluconazole resistance (Ni et al., 2013). C. neoformans can also form “titan” cells, which are larger than normal haploid cells. These cells are frequently tetraploid (4N), octoploid (8N), or even higher ploidy. Under stressful conditions, these titan cells can produce near diploid daughter cells, with aneuploidies affecting different chromosomes. These aneuploid cells often show resistance to different drugs. Chr I aneuploidy was found to occur more often (in 26 out of 53 strains), but other chromosomes also assisted with fluconazole resistance (Gerstein et al., 2015)

#### 2.3.4 Leishmania and Trypanozoma

As a side note, adaptation through aneuploidy is also known to occur in nonfungal pathogenic microbes. Trypanosomatids, including *Leishmania* and *Trypanozoma*, are responsible for diseases such as sleeping sickness (*Trypanosoma* brucei) and Chagas disease (*Trypanosoma* cruzi). Aneuploidy is particularly widespread across *Leishmania* and has been shown to vary from few aneuploid chromosomes (Valdivia et al., 2017) to many (Coughlan et al., 2017; Coughlan et al., 2018; Iantorno et al., 2017; Kumar et al., 2013; Prieto Barja et al., 2017; Reis‐Cunha et al., 2015; Rogers et al., 2011; Ubeda et al., 2008) depending on the species, with large phenotypic effects, including resistance to various drugs. For example, *Leishmania* major gains resistance to the drugs methotrexate and antimonial through aneuploidy, *Leishmania* donovani to the drug nelfinavi and *Leishmania infantum* to methotrexate (Kumar et al., 2013; Mukherjee et al., 2013; Ubeda et al., 2008). Aneuploidy has also been shown to result in reduced generation time, increasing fitness in vitro in L. donovani, where chromosome copy number in aneuploids was positively correlated with gene expression in all but one chromosome, that is, an extra copy of a chromosome caused gene expression of most genes on that chromosome to double (testing effects of genomic coverage on transcriptomic coverage: *r* = 0.72; Prieto Barja et al., 2017). In T. cruzi, several studies have noted the presence of aneuploidy (Lewis et al., 2009; Minning, Weatherly, Flibotte, & Tarleton, 2011), which appears to vary between strains (Reis‐Cunha et al., 2015), but to our knowledge, these aneuploidies have not been linked with aspects of pathogenicity or drug resistance yet.

## 3 PATTERNS OF ANEUPLOIDY—CHROMOSOME SIZE, PLOIDY, AND THE ENVIRONMENT

Although the occurrence of aneuploidy, like any mutation, is stochastic, certain patterns emerge. Here, we investigate associations between rates of aneuploidy, initial average population ploidy and chromosome size.

### 3.1 Initial ploidy and rates of aneuploidy

**Figure 3:**
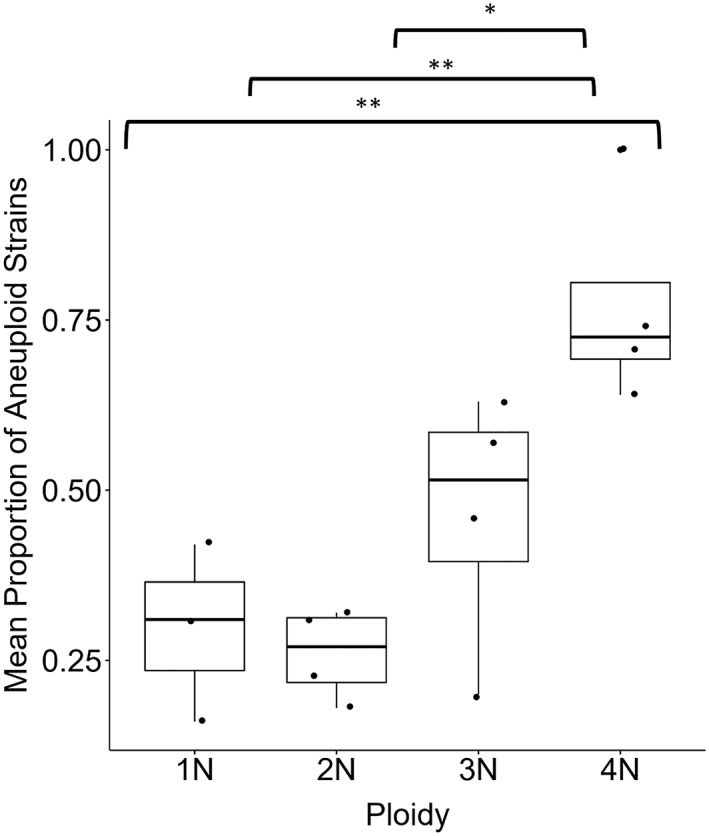
Proportion of aneuploid to euploid strains as a function of ploidy. Data was extracted from four sources (Duan et al., 2018; Gallone et al., 2016; Peter et al., 2018; Zhu et al., 2016) with a total of 1,547 strains between them (158 haploid, 1,149 diploid, 104 triploid, and 131 tetraploid). The proportion of aneuploid to euploid strains was calculated per study, means and standard errors were calculated across studies. Analyses and graphs were made in R version 3.5.1 (Feather Spray; R Core Team, 2018) with the packages ggplot2 (Wickham, 2016), ggpubr (Kassambara, 2018), and magrittr (Bache & Wickham, 2014). (Kruskal–Wallis χ ^2^ = 9.86, p = .02, pairwise t‐test [4N] p < .05. ^*^ p < 0.05, ^**^ p < .01)

The ploidy of a population determines, to an extent, its chances to lose or gain chromosomes and thus its predisposition to turn aneuploid. Our analysis of published data from four independent studies, encompassing 1,547 strains (Duan et al., 2018; Gallone et al., 2016; Peter et al., 2018; Zhu et al., 2016), revealed a general pattern of increasing proportion of aneuploid strains with increasing ploidy (Kruskal–Wallis *χ* ^2^ = 9.86, *p* = .02, *df* = 3), with tetraploid strains containing significantly higher rates of aneuploidy than lower ploidy strains (*t* = 2.2, all *p* ≤ .05; Figure 3 and Table S3). This may simply be a result of the circumstance that in genomes with higher ploidy, chances for aneuploidy much higher, especially when both chromosome gains and losses are considered. Alternatively, higher ploidies may cause additional genomic instability, perhaps due to higher mutation rates or more frequent chromosome loss, affecting some chromosomes more than others. For instance, Todd et al. (2017) report tetraploid S. cerevisiae cells to have a 200‐ to 1,000‐fold increase in the rate of chromosome loss compared with isogenic diploid cells. This effect is also well known from cancer genetics where tetraploidy promotes chromosomal aberrations and tumourigenesis (Ganem, Storchova, & Pellman, 2007; Lv et al., 2012).

Further evidence comes from experimental case studies observing that under relaxed selection, diploid S. cerevisiae populations gained significantly more chromosomes than haploid populations (binomial test: *p* < 10^−10^; Sharp, Sandell, James, & Otto, 2018). Another study, which compared haploids with populations that had undergone whole genome duplication (WGD) to become diploid, found aneuploidy exclusively in the diploid populations (Fisher, Buskirk, Vignogna, Marad, & Lang, 2018). A further study found that haploid and diploid strains were significantly less likely to contain aneuploid chromosomes than >3N populations (Fisher's exact test *p* < .001; *n* of strains = 142, (Zhu et al., 2016). Finally, in response to low carbon conditions, tetraploid (4N) populations of S. cerevisiae were the only populations found to contain aneuploidy, with haploid and diploid populations remaining euploid. Most of these were loss‐of‐chromosome events,but gains of Chr XIII were also observed significantly more times than any other chromosome (Cochran–Armitage test, *p* < 1 × 10^−7^, *n* = 100; Selmecki et al., 2015). Interestingly, an extra copy of Chr XIII in diploid populations was detrimental, highlighting the possibility that these mutational effects are ploidy specific.

### 3.2 Chromosome size and rates of aneuploidy

There is some debate as to whether organisms are able to tolerate aneuploidy better in smaller chromosomes, with increased fitness costs potentially associated with larger chromosomes. In *Saccharomyces* yeasts, several studies have found a significant negative correlation between chromosome size and rate of aneuploidy (Kumaran, Yang, & Leu, 2013; Peter et al., 2018; Zhu et al., 2016). There have also been references to this pattern in studies of C. albicans (Forche et al., 2018) and C. neoformans var. *grubii* (Rhodes et al., 2017). This suggests that this pattern is not unique to S. cerevisiae due to their unconventional point centromere structure (Furuyama & Biggins, 2007).

**Figure 4:**
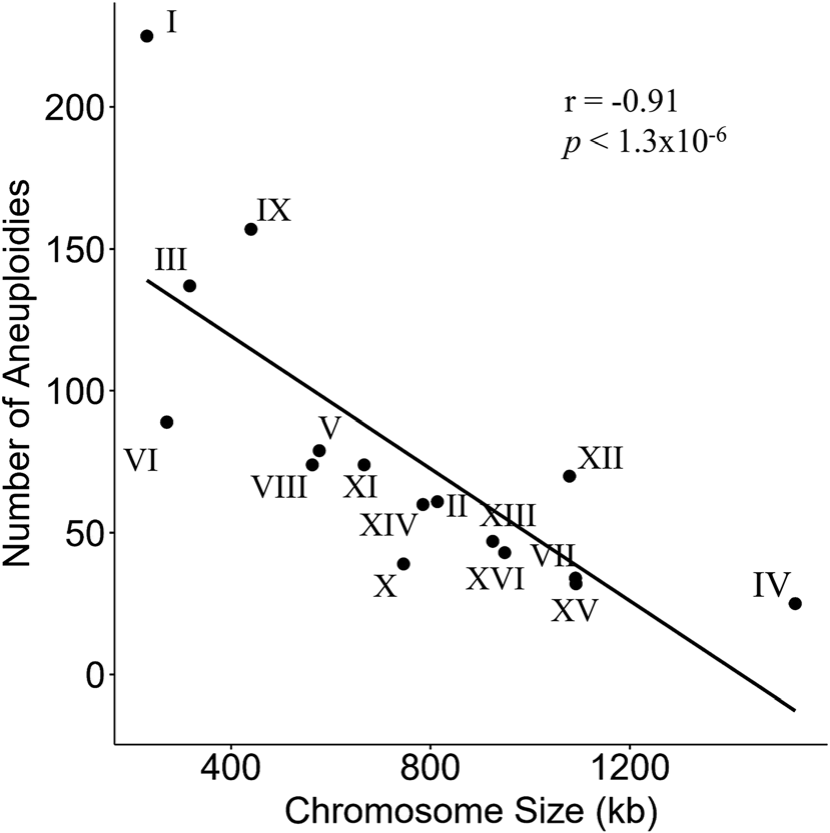
Number of aneuploidy incidents as a function of chromosome size. Data was extracted from eight sources (Duan et al., 2018; Gallone et al., 2016; Jaffe et al., 2017; Kao et al., 2010; Peter et al., 2018; Selmecki et al., 2015; Sharp et al., 2018; Zhu et al., 2016), with a total of 1,207 aneuploidies between them. Chromosomes are labelled with Roman numerals

We tested for a relationship between chromosome size and the frequency of aneuploidy in S. cerevisiae using published data from eight different studies, with 1,228 aneuploidies between them (Duan et al., 2018; Gallone et al., 2016; Jaffe, Sherlock, & Levy, 2017; Kao et al., 2010; Peter et al., 2018; Selmecki et al., 2015; Sharp et al., 2018; Zhu et al., 2016; Table S1). We found a significant negative correlation between aneuploidy and chromosome size (Spearman's rank correlation: *r* = −0.91, *p* < 1.3 × 10^−6^, *df* = 14; Figure 4). Figure 4 reveals some interesting exceptions to the above pattern. For instance, despite being the second smallest, Chr VI shows an unexpectedly low incident of aneuploidies across studies. Disomy of Chr VI was previously suggested as lethal on its own, due to the genes TUB2 (involved in tubulin formation) and ACT1 (part of the cytoskeleton; Torres et al., 2007), both of which reduce cell viability when expression is increased (Liu, Krizek, & Bretscher, 1992; Weinstein & Solomon, 1990). It was suggested that aneuploidy of Chr VI may be better tolerated in combination with other aneuploidies, for example, Chr I and Chr XIII (Torres et al., 2007). A study of stability of aneuploidies in S. cerevisiae also found few examples of aneuploidy in Chr VI, with any incidences categorised as highly unstable (Zhu et al., 2012). Interestingly, aneuploidies of chromosome VI have been shown to evolve as a rescue mechanism in response to selection for the compensation of deleterious mutations (Filteau et al., 2015). This shows that although size of chromosome is important in predicting chance of aneuploidy, other factors, for example, environmental or genetic constraints, also need to be considered (Box [Link] provides more information).

The studies we considered here can be roughly divided into laboratory experiments using specific strains of S. cerevisiae (recording 332 aneuploidies in total; Table S2D; Jaffe et al., 2017; Kao et al., 2010; Selmecki et al., 2015; Sharp et al., 2018) and nonlaboratory studies (recording 914 aneuploidies in total; Duan et al., 2018; Gallone et al., 2016; Peter et al., 2018; Zhu et al., 2016). We separated strains from nonlaboratory studies into three further categories to test for a relationship between chromosome size and the frequency of aneuploidy: industrial (*n* = 604; Table S2A; Duan et al., 2018; Gallone et al., 2016; Peter et al., 2018), pathogenic (*n* = 146; Table S2B; Peter et al., 2018; Zhu et al., 2016), and wild strains (*n* = 146; Table S2C; Duan et al., 2018; Peter et al., 2018), with some remaining uncategorised (Peter et al., 2018). All three nonlaboratory strain types showed significant negative correlations between aneuploidy and chromosome size (industrial populations Spearman's rank correlation: *r* = −0.83, *p* = 6.18 × 10^−5^, *df* = 14, Figure S1A; pathogenic populations Spearman's rank correlation: *r* = −0.64, *p* = .008, *df* = 14, Figure S1B; wild populations Spearman's rank correlation: *r* = −0.59, *p* = .02, *df* = 14, Figure S1C).

Data from laboratory studies alone did not show a strong relationship between aneuploidy and chromosome size (Pearson's *r* = −0.47, *p* = .07, *df* = 14, Figure S1D). Although this may simply be due to a lack of statistical power, one possible explanation is that the experimental treatments used in these studies were so stressful that aneuploidies even of larger chromosomes have net fitness benefits. For example, selection for aneuploidy of the large chromosome XIII has been observed under low carbon conditions (Selmecki et al., 2015).

### 3.3 Aneuploidy patterns in specific environments

Three aspects of stress crucially determine if adaptation through aneuploidy occurs at all, and which aneuploidy occurs: (a) the type of stress, (b) the degree of stress, and (c) the rate at which it is applied. For example, aneuploidy in S. cerevisiae populations was only observed when subjected abruptly to high levels of stress. A more gradual increase in stress resulted in no aneuploidy (Yona et al., 2012). Interestingly, another study comparing gradual versus abrupt changes in heavy metal stress (cadmium) found the opposite pattern; aneuploidy events occurred most commonly in gradually increasing concentrations of cadmium (Gorter et al., 2017). This suggests that different types of stress trigger different aneuploidy responses. Box [Link] provides a summary of what is known about the adaptive potential of aneuploidy when cells face different types of stress, such as temperature, starvation, toxic stress, and telomerase insufficiency (see also Table 1).

## 4 HYBRIDISATION AND ANEUPLOIDY THROUGH MEIOSIS

Hybrid meiosis, especially when involving genetically divergent species, can cause high aneuploidy rates among the F1 offspring. This is well studied in the budding yeast hybrids S. cerevisiae × S. paradoxus, which differ at about 14% of all nucleotides in their genomes (Liti et al., 2009). When crossed, a large proportion of their first generation hybrids (F1) produce aneuploid spores. Rogers et al. recently showed that of ~15,000 F1 hybrid tetrads studied, more than a third exhibited nondisjunction. Spores carried at least one aneuploidy (40.3% per chromosome across all spores) with between one and five chromosomes affected per spore (Rogers et al., 2018). The underlying molecular mechanism causing infertility between species is most likely antirecombination, where a lack of recombination caused by the mismatch repair (MMR) system causes missegregation of homeologous chromosomes during interspecific hybrid meiosis (Chambers, Hunter, Louis, & Borts, 1996; Greig, 2009; Hunter, Chambers, Louis, & Borts, 1996; Kao et al., 2010). Further evidence for this comes from the deletion of two genes involved in MMR (PMS1 and MSH2), which both independently reduce the rate of aneuploidy in hybrids and increase spore viability (Hunter et al., 1996; Kao et al., 2010).

To our knowledge, and after thorough reviewing of the literature, little data exist on the role that aneuploidy plays in the adaptation of hybrids and the relationships remain correlative. The best‐studied case are probably the interspecific hybrids between C. neoformans × *Cryptococcus gattii* (serotype AD) frequently found in the wild (Cuomo, Rhodes, & Desjardins, 2018; Li et al., 2012). Four out of 10 of these hybrid strains showed signs of aneuploidy, with DNA content between haploid and diploid levels (Lengeler et al., 2001). Two out of three particularly virulent hybrid AD strains isolated from AIDS patients contained an extra Chr XIII (Hu et al., 2008), which was linked with the increased virulence of these strains (Hu et al., 2011). Another study measuring the fitness of C. neoformans × *C. gattii* hybrids (but not their ploidy) in 40 different environments found extensive transgressive segregation (i.e., hybrids were fitter than the parents), suggesting that hybridisation may play a significant role in the phenotypic evolution of this important human pathogen (Shahid, Han, Yoell, & Xu, 2008).

In a study of 212 predominantly industrial strains used in wine and beer fermentation, 17 strains were identified to be hybrids, of which a third (six strains) showed signs of aneuploidy (Borneman, Forgan, Kolouchova, Fraser, & Schmidt, 2016). Ten of the 17 hybrids strains had contributions from the distantly related yeast species *Saccharomyces kudriavzevii*. In fact, S. cerevisiae × *S. kudriavzevii* hybrids often play a role in wine fermentation, mostly unintentionally (Belloch et al., 2009; González, Barrio, Gafner, & Querol, 2006; Lopandic et al., 2007). These hybrids often show elevated DNA content indicative of aneuploidy (Belloch et al., 2009; Erny et al., 2012). For instance, the S. cerevisiae × *S. kudriavzevii* hybrid strain Eg8 has been shown to contain a diploid set of S. cerevisiae and a haploid set of *S. kudriavzevii* chromosomes. However, due to various aneuploidies individual chromosomes from S. cerevisiae vary from between one to four copies across all 17 isolates tested (Erny et al., 2012). This strain is thought to have originally formed due to hybridisation between a diploid S. cerevisiae spore and a haploid *S. kudriavzevii* spore.

Interestingly, the pattern of decreasing aneuploidy rates as chromosome size increases (Figure 4) has been reported in meiotic products of intraspecific S. paradoxus × S. paradoxus hybrids made from divergent strains (N17 × N44; Rogers et al., 2018). This was not observed in meiotic products of interspecific S. cerevisiae × S. paradoxus hybrids (Rogers et al., 2018). To uncover the exact mechanism behind this discrepancy will require further experimentation. If we were to speculate, as the number of crossovers in S. cerevisiae directly scales with chromosome size (Mancera, Bourgon, Brozzi, Huber, & Steinmetz, 2008), this could explain why smaller chromosomes are more likely to become aneuploid in intraspecific hybrids (Rogers et al., 2018). However, this does not explain why the pattern is absent in interspecific hybrids. Interspecific hybrids often experience antirecombination, resulting in reduced crossing over due to the MMR mechanism. If this reduces crossing over in all chromosomes, it is possible that antirecombination would cause a similar pattern. One way to test this would be to use a combination of MMR deficient strains, for example, MSH2 knockout strains (Kao et al., 2010), and to measure aneuploidy rates in both viable and inviable spores, for example, using fluorescence markers. If deficiency in the MMR pathway in interspecific hybrids reinstated the pattern of chromosome size and rate of aneuploidy seen in intraspecific hybrids, then this may be the underlying mechanism. This could help uncover the discrepancy, as well as reinforce the role of antirecombination in hybrid sterility.

Of course, none of the above studies presents direct evidence that aneuploidy can promote adaptation in hybrids. However, it is reasonable to expect that aneuploidy in hybrids can bring about competitive advantages, using the same genetic and molecular mechanisms as in nonhybrid strains. In addition, consider the following circumstances. Small isolated populations existing at the margins of species ranges are more likely to hybridise with neighbouring, genetically divergent populations or species (McFarlane & Pemberton, 2019). Hybridisation should automatically increase the frequency of aneuploids in these environments (whether aneuploidy is beneficial or not; Rogers et al., 2018). At the same time, populations existing at the periphery where ecological conditions are more challenging are more likely to experience stress (Macdonald, Llewelyn, Moritz, & Phillips, 2017). We suggest that the convergence of these processes—hybridisation and aneuploidy—and the increased chance for them to occur at the same time and place under environmental stress may give aneuploid hybrid genomes a transient solution to adaptation. Indeed, aneuploid hybrids may be more stress tolerant as a result of more flexible gene dosage compensation. This could facilitate the evolution of hybrid microbial populations with new ecological characteristics (e.g., new drug resistance or increased infectivity) in habitats where the parental strains would not be able to survive.

## 5 CONCLUDING REMARKS

Under conditions where populations require rapid adaptive responses to a detrimental environment to be able to survive, aneuploidy through chromosomal nondisjunction during mitosis can provide a quick solution allowing for adaptation. In cancer cells, for instance, aneuploidy is known to result in deregulation of the transcriptome and proteome (Dürrbaum & Storchová, 2016; Ried et al., 2012). Aneuploidy, as with any other alteration in gene copy number (Lauer et al., 2018), can result in differential gene expression (Sheltzer, Torres, Dunham, & Amon, 2012). Under certain conditions, such changes in gene dosage may provide fitness benefits to the cell that outweigh the costs of maintaining the aneuploidy. This includes drug resistance, adaptation to environmental stress and, in pathogenic taxa, the colonisation of novel hosts and higher virulence, which in turn can promote their evolutionary diversification (Figure 1; Selmecki et al., 2006; Selmecki et al., 2015; Yona et al., 2012).

Specific chromosomes are more often affected by aneuploidy than others in response to specific types of stress (Figure 4; Box [Link]). These patterns of aneuploidy are interesting because they may allow for the identification of genes or regions involved in stress response (e.g., several genes on Chr III provide heat tolerance; Yona et al., 2012). This can be informative for industrial applications, for example, for the engineering of strains that are more efficient bioethanol producers, or strains resistant to radiation, heavy metal, or acid to clean up the toxic environments that are human waste products. This knowledge could also be helpful to improve the treatment of infections, for example, by avoiding certain drugs known to induce aneuploidy and therefore drug resistance. For example, the aneuploid daughters of C. neoformans titan cells provide a good target for study of aneuploidy and its impacts on pathogenicity.

Our analysis and that of others (reviewed in Todd et al., 2017) show that S. cerevisiae strains with higher initial ploidy are also generally more likely to be aneuploid (Figure 3). WGD has played an important role in the diversification and evolution of *Saccharomyces* yeast (Albertin et al., 2009). It is thus conceivable that in the process of WGD or polyploidisation in general, aneuploidy is a regular, accidental by‐product. We think it is therefore possible that over the course of the merging and diverging of yeast species, including several independent WGD events, aneuploidy may have featured many times in the natural history of yeasts. We suggest that in a similar manner to the sub‐ or neo‐functionalization of genomic regions known to occur after WGD events (Van de Peer, Mizrachi, & Marchal, 2017), having an extra copy of a chromosome, even temporarily, may allow for an increased evolvability of the genome.

A conclusive demonstration of aneuploidy playing any formative role in macroevolutionary processes is of course a tall order, especially due to the transient nature of aneuploidy. Experimental evolution studies have shown that changes in gene dosage leading to phenotypic innovations due to aneuploidy will only be based on the effects of the extra chromosome for a limited number of generations, until it is shed from the cell and replaced by less cumbersome, more stable genomic solutions (Yona et al., 2012). So even if aneuploidy is an important driver of adaptation to changing environments, and wild populations isolated from stressful environments have gained or lost chromosomes sometime in the past, environmental sequencing of wild strains is likely to underestimate aneuploidy as once in the laboratory, exposure to an overabundance of nutrients and absence of environmental stress could cause a quick loss of extra chromosomes.

We suggest future studies could use next generation sequencing to investigate signatures of past aneuploidy in the genome. One approach may be to exploit the fact that even temporary aneuploidy causes the affected chromosome to be a more likely target for selection due to its higher copy number. The affected chromosome may carry the “ghost of an aneuploid past,” perhaps in the form of higher evolutionary rates as aneuploidy, much like gene duplication, frees up genes on the extra chromosomal copies for taking on new functions by mutation. It is intriguing to think about the dynamics ensuing from different genetic architectures of adaptive traits in this context. For instance, would aneuploidy be more or less likely retained if adaptation was based on a few genes located on the extra chromosome, as compared with a trait with a quantitative genetic basis that has many genes on the extra chromosome?

It has been demonstrated experimentally that interspecific S. cerevisiae *× S. paradoxus* yeast hybrids can colonise new ecological niches (Stelkens, Brockhurst, Hurst, & Greig, 2014; Stelkens, Brockhurst, Hurst, Miller, & Greig, 2014), and that S. cerevisiae *× Saccharomyces uvarum* hybrids can gain resistance to stress through genomic rearrangements such as chromosomal fusions and translocations (Dunn et al., 2013; Piotrowski et al., 2012). There is no data to date on the long‐term benefits or costs of aneuploidy through hybrid meiosis. However, we think this is a fruitful field for further research, given (a) the accumulating evidence for adaptation through aneuploidy (Selmecki et al., 2015; Yona et al., 2012), (b) the evidence that hybrid meiosis leads to high rates of aneuploidy in yeast (Rogers et al., 2018), and (c) the circumstance that both aneuploidy and hybridisation occur in stressful and perturbed habitats (Garroway et al., 2010; Muhlfeld et al., 2014). Whether hybridisation will generally assist or hamper adaptation to changing environments, with or without aneuploidy, is itself a debated topic (Hamilton & Miller, 2016; Kovach, Luikart, Lowe, Boyer, & Muhlfeld, 2016; Miller & Hamilton, 2016). In pathogenic microbes, this has particular relevance due to the medical risk of new hybrid pathogens emerging through genetic exchange with potentially increased virulence, larger host ranges, or increased drug resistance (Ochman, Lawrence, & Groisman, 2000).

A combination of available and new molecular and phenotypic strain typing, microscopy, and karyotyping techniques should soon allow for a more in‐depth understanding of aneuploidy and its evolutionary significance.

## CONFLICT OF INTEREST

The authors declare no conflict of interest.

## Supporting information

Table S1.
Table of all aneuploidies used for analysis of chromosome size and rate of aneuploidy (Figure 2). This includes aneuploidies reported by each reference, as well as the total aneuploidies, ordered by size of chromosome.

Table S2
Tables containing the aneuploidies used for analysis of chromosomes size and rate of aneuploidy separated into four categories. A) Industrial strains (Supplementary Figure 1A), B) pathogenic strains (Supplementary Figure 1B), C) wild strains (Supplementary Figure 1C) and experimental strains (Supplementary Figure 1D). This includes aneuploidies reported by each reference, as well as the total aneuploidies, ordered by chromosome size. Industrial strains include those linked with beer, wine, other fermentation and bioethanol production. Pathogenic strains are those found in clinical and other human samples. Wild strains are those found on plants, in insects, in water and other natural settings. Experimental strains are classified as strains involved in laboratory experiments, in which strains start as clonal and are studied over time. This includes a hybridisation experiment, studies of a deletion collection and studies of adaptation to starvation. 18 strains were uncategorised and are not included in these tables. These uncategorised strains are included in Supplementary Table 1 and Figure 2.

Table S3.
Table containing the data used for analysis of ploidy and proportion of aneuploid strains (Figure 4). Proportions are separated by individual references and the mean of all proportions for each ploidy.

Figure S1.
Scatter plots of the data from Supplementary Tables 2. This includes industrial strains (A), pathogenic strains (B), wild strains (C) and experimental strains (D). Regression line indicates significant correlation. Analyses and graphs were made using R version 3.5.1 (Feather Spray; R Core Team, 2018) with the packages ggplot2 (Wickham, 2016), ggpubr (Kassambara, 2018) and magrittr (Bache & Wickham, 2014).

## References

[yea3427-bib-0001] Adamczyk, J. , Deregowska, A. , Potocki, L. , Kuna, E. , Kaplan, J. , Pabian, S. , … Wnuk, M. (2016). Relationships between rDNA, Nop1 and Sir complex in biotechnologically relevant distillery yeasts. Archives of Microbiology, 198, 715–723. 10.1007/s00203-016-1258-9 27329282 PMC4969353

[yea3427-bib-0002] Ahmad, K. M. , Ishchuk, O. P. , Hellborg, L. , Jørgensen, G. , Skvarc, M. , Stenderup, J. , … Piškur, J. (2013). Small chromosomes among Danish *Candida glabrata* isolates originated through different mechanisms. Antonie Van Leeuwenhoek, 104, 111–122. 10.1007/s10482-013-9931-3 23670790 PMC3672514

[yea3427-bib-0003] Albertin, W. , Marullo, P. , Aigle, M. , Bourgais, A. , Bely, M. , Dillmann, C. , … Sicard, D. (2009). Evidence for autotetraploidy associated with reproductive isolation in *Saccharomyces cerevisiae*: Towards a new domesticated species. Journal of Evolutionary Biology, 22, 2157–2170. 10.1111/j.1420-9101.2009.01828.x 19765175

[yea3427-bib-0004] Bache SM , Wickham H . 2014 magrittr: A forward‐pipe operator for R. https://cran.r‐project.org/package=magrittr.

[yea3427-bib-0005] Bader, O. , Schwarz, A. , Kraneveld, E. A. , Tangwattanchuleeporn, M. , Schmidt, P. , Jacobsen, M. D. , … Weig, M. (2012). Gross karyotypic and phenotypic alterations among different progenies of the *Candida glabrata* CBS138/ATCC2001 reference strain. PLoS ONE, 7, e52218 10.1371/journal.pone.0052218 23284942 PMC3527424

[yea3427-bib-0006] Beach, R. R. , Ricci‐Tam, C. , Brennan, C. M. , Moomau, C. A. , Hsu, P. , Hua, B. , … Amon, A. (2017). Aneuploidy causes non‐genetic individuality. Cell, 169, 229–242.e21. 10.1016/j.cell.2017.03.021 28388408 PMC5441241

[yea3427-bib-0007] Belloch, C. , Perez‐Torrado, R. , Gonzalez, S. S. , Perez‐Ortin, J. E. , Garcia‐Martinez, J. , Querol, A. , & Barrio, E. (2009). Chimeric genomes of natural hybrids of *Saccharomyces cerevisiae* and *Saccharomyces kudriavzevii* . Applied and Environmental Microbiology, 75, 2534–2544. 10.1128/AEM.02282-08 19251887 PMC2675219

[yea3427-bib-0008] Bennett, R. J. , & Johnson, A. D. (2003). Completion of a parasexual cycle in *Candida albicans* by induced chromosome loss in tetraploid strains. The EMBO Journal, 22, 2505–2515. 10.1093/emboj/cdg235 12743044 PMC155993

[yea3427-bib-0009] Biesecker, L. G. , & Spinner, N. B. (2013). A genomic view of mosaicism and human disease. Nature Reviews Genetics, 14, 307–320. 10.1038/nrg3424 23594909

[yea3427-bib-0010] Borneman, A. R. , Forgan, A. H. , Kolouchova, R. , Fraser, J. A. , & Schmidt, S. A. (2016). Whole genome comparison reveals high levels of inbreeding and strain redundancy across the spectrum of commercial wine strains of *Saccharomyces cerevisiae* . G3: Genes|Genomes|Genetics, 6, 957–971. 10.1534/g3.115.025692 26869621 PMC4825664

[yea3427-bib-0011] Boynton, P. J. , Janzen, T. , & Greig, D. (2018). Modeling the contributions of chromosome segregation errors and aneuploidy to *Saccharomyces* hybrid sterility. Yeast, 35, 85–98. 10.1002/yea.3282 28967670

[yea3427-bib-0012] Chambers, S. R. , Hunter, N. , Louis, E. J. , & Borts, R. H. (1996). The mismatch repair system reduces meiotic homeologous recombination and stimulates recombination‐dependent chromosome loss. Molecular and Cellular Biology, 16, 6110–6120. 10.1128/MCB.16.11.6110 8887641 PMC231614

[yea3427-bib-0013] Chang, S.‐L. , Lai, H.‐Y. , Tung, S.‐Y. , & Leu, J.‐Y. (2013). Dynamic large‐scale chromosomal rearrangements fuel rapid adaptation in yeast populations. PLoS Genetics, 9, e1003232 10.1371/journal.pgen.1003232 23358723 PMC3554576

[yea3427-bib-0014] Chen, G. , Bradford, W. D. , Seidel, C. W. , & Li, R. (2012). Hsp90 stress potentiates rapid cellular adaptation through induction of aneuploidy. Nature, 482, 246–250. 10.1038/nature10795 22286062 PMC3276732

[yea3427-bib-0015] Chen, G. , Mulla, W. A. , Kucharavy, A. , Tsai, H. J. , Rubinstein, B. , Conkright, J. , … Li, R. (2015). Targeting the adaptability of heterogeneous aneuploids. Cell, 160, 771–784. 10.1016/j.cell.2015.01.026 25679766 PMC4328141

[yea3427-bib-0016] Coughlan, S. , Mulhair, P. , Sanders, M. , Schonian, G. , Cotton, J. A. , & Downing, T. (2017). The genome of *Leishmania adleri* from a mammalian host highlights chromosome fission in Sauroleishmania. Scientific Reports, 7, 43747 10.1038/srep43747 28256610 PMC5335649

[yea3427-bib-0017] Coughlan, S. , Taylor, A. S. , Feane, E. , Sanders, M. , Schonian, G. , Cotton, J. A. , & Downing, T. (2018). *Leishmania naiffi* and *Leishmania guyanensis* reference genomes highlight genome structure and gene evolution in the Viannia subgenus. Royal Society Open Science, 5, 172212 10.1098/rsos.172212 29765675 PMC5936940

[yea3427-bib-0018] Cox, B. S. , & Bevan, E. A. (1962). Aneuploidy in yeast. New Phytologist, 61, 342–355. 10.1111/j.1469-8137.1962.tb06305.x

[yea3427-bib-0019] Cuomo, C. A. , Rhodes, J. , & Desjardins, C. A. (2018). Advances in *Cryptococcus* genomics: Insights into the evolution of pathogenesis. Memórias do Instituto Oswaldo Cruz, 113, 1–7. http://www.scielo.br/scielo.php?script=sci_arttext&pid=S0074‐02762018000700201&lng=en&tlng=en 29513784 10.1590/0074-02760170473 PMC5851040

[yea3427-bib-0020] Deregowska, A. , Skoneczny, M. , Adamczyk, J. , Kwiatkowska, A. , Rawska, E. , Skoneczna, A. , … Wnuk, M. (2015). Genome‐wide array‐CGH analysis reveals YRF1 gene copy number variation that modulates genetic stability in distillery yeasts. Oncotarget, 6, 30650–30663. http://www.ncbi.nlm.nih.gov/pubmed/26384347, 10.18632/oncotarget.5594 26384347 PMC4741559

[yea3427-bib-0021] Duan, S.‐F. , Han, P.‐J. , Wang, Q.‐M. , Liu, W.‐Q. , Shi, J.‐Y. , Li, K. , … Bai, F.‐Y. (2018). The origin and adaptive evolution of domesticated populations of yeast from Far East Asia. Nature Communications, 9, 2690 10.1038/s41467-018-05106-7 PMC6043522 30002370

[yea3427-bib-0022] Dunlap, S. S. , Aziz, M. A. , & Rosenbaum, K. N. (1986). Comparative anatomical analysis of human trisomies 13, 18, and 21: I. The forelimb. Teratology, 33, 159–186. 10.1002/tera.1420330204 2943045

[yea3427-bib-0023] Dunn, B. , Paulish, T. , Stanbery, A. , Piotrowski, J. , Koniges, G. , Kroll, E. , … Rosenzweig, F. (2013). Recurrent rearrangement during adaptive evolution in an interspecific yeast hybrid suggests a model for rapid introgression. PLoS Genetics, 9, e1003366 10.1371/journal.pgen.1003366 23555283 PMC3605161

[yea3427-bib-0024] Dunn, B. , & Sherlock, G. (2008). Reconstruction of the genome origins and evolution of the hybrid lager yeast *Saccharomyces pastorianus* . Genome Research, 18, 1610–1623. 10.1101/gr.076075.108 18787083 PMC2556262

[yea3427-bib-0025] Dürrbaum, M. , & Storchová, Z. (2016). Effects of aneuploidy on gene expression: Implications for cancer. FEBS Journal, 283, 791–802. 10.1111/febs.13591 26555863

[yea3427-bib-0026] Erny, C. , Raoult, P. , Alais, A. , Butterlin, G. , Delobel, P. , Matei‐Radoi, F. , … Legras, J. L. (2012). Ecological success of a group of *Saccharomyces cerevisiae*/*Saccharomyces kudriavzevii* hybrids in the northern European wine‐making environment. Applied and Environmental Microbiology, 78, 3256–3265. 10.1128/AEM.06752-11 22344648 PMC3346444

[yea3427-bib-0027] Filteau, M. , Hamel, V. , Pouliot, M.‐C. , Gagnon‐Arsenault, I. , Dube, A. K. , & Landry, C. R. (2015). Evolutionary rescue by compensatory mutations is constrained by genomic and environmental backgrounds. Molecular Systems Biology, 11, 832–832. 10.15252/msb.20156444 26459777 PMC4631203

[yea3427-bib-0028] Fisher, K. J. , Buskirk, S. W. , Vignogna, R. C. , Marad, D. A. , & Lang, G. I. (2018). Adaptive genome duplication affects patterns of molecular evolution in *Saccharomyces cerevisiae* . PLoS Genetics, 14, e1007396 10.1371/journal.pgen.1007396 29799840 PMC5991770

[yea3427-bib-0029] Forche, A. , Cromie, G. , Gerstein, A. C. , Solis, N. V. , Pisithkul, T. , Srifa, W. , … Berman, J. (2018). Rapid phenotypic and genotypic diversification after exposure to the oral host niche in *Candida albicans* . Genetics, 209, 725–741. 10.1534/genetics.118.301019 29724862 PMC6028260

[yea3427-bib-0030] Forche, A. , Magee, P. T. , Selmecki, A. , Berman, J. , & May, G. (2009). Evolution in *Candida albicans* populations during a single passage through a mouse host. Genetics, 182, 799–811. 10.1534/genetics.109.103325 19414562 PMC2710160

[yea3427-bib-0031] Furuyama, S. , & Biggins, S. (2007). Centromere identity is specified by a single centromeric nucleosome in budding yeast. Proceedings of the National Academy of Sciences, 104, 14706–14711. 10.1073/pnas.0706985104 PMC1976213 17804787

[yea3427-bib-0032] Gallone, B. , Steensels, J. , Prahl, T. , Soriaga, L. , Saels, V. , Herrera‐Malaver, B. , … Verstrepen, K. J. (2016). Domestication and divergence of *Saccharomyces cerevisiae* beer yeasts. Cell, 166, 1397–1410.e16. 10.1016/j.cell.2016.08.020 27610566 PMC5018251

[yea3427-bib-0033] Ganem, N. J. , Storchova, Z. , & Pellman, D. (2007). Tetraploidy, aneuploidy and cancer. Current Opinion in Genetics & Development, 17, 157–162. http://linkinghub.elsevier.com/retrieve/pii/S0959437X07000378, 10.1016/j.gde.2007.02.011 17324569

[yea3427-bib-0034] Gao, C. , Furge, K. , Koeman, J. , Dykema, K. , Su, Y. , Cutler, M. L. , … Vande Woude, G. F. (2007). Chromosome instability, chromosome transcriptome, and clonal evolution of tumor cell populations. Proceedings of the National Academy of Sciences, 104, 8995–9000. 10.1073/pnas.0700631104 PMC1885616 17517657

[yea3427-bib-0035] Gao, C. , Su, Y. , Koeman, J. , Haak, E. , Dykema, K. , Essenberg, C. , … Vande Woude, G. F. (2016). Chromosome instability drives phenotypic switching to metastasis. Proceedings of the National Academy of Sciences, 113, 14793–14798. 10.1073/pnas.1618215113 PMC5187712 27930335

[yea3427-bib-0036] Garroway, C. J. , Bowman, J. , Cascaden, T. J. , Holloway, G. L. , Mahan, C. G. , Malcolm, J. R. , … Wilson, P. J. (2010). Climate change induced hybridization in flying squirrels. Global Change Biology, 16, 113–121. 10.1111/j.1365-2486.2009.01948.x

[yea3427-bib-0037] Gerstein, A. C. , Fu, M. S. , Mukaremera, L. , Li, Z. , Ormerod, K. L. , Fraser, J. A. , … Nielsen, K. (2015). Polyploid titan cells produce haploid and aneuploid progeny to promote stress adaptation. MBio, 6, e01340–e01315. 10.1128/mBio.01340-15 26463162 PMC4620463

[yea3427-bib-0038] González, S. S. , Barrio, E. , Gafner, J. , & Querol, A. (2006). Natural hybrids from *Saccharomyces cerevisiae*, *Saccharomyces bayanus* and *Saccharomyces kudriavzevii* in wine fermentations. FEMS Yeast Research, 6, 1221–1234. 10.1111/j.1567-1364.2006.00126.x 17156019

[yea3427-bib-0039] Gorter de Vries, A. R. , Pronk, J. T. , & Daran, J.‐M. G. (2017). Industrial relevance of chromosomal copy number variation in *Saccharomyces* yeasts. Applied and Environmental Microbiology, 83: AEM, 03206–03216. 10.1128/AEM.03206-16 PMC5440705 28341679

[yea3427-bib-0040] Gorter, F. A. , Derks, M. F. L. , van den Heuvel, J. , Aarts, M. G. M. , Zwaan, B. J. , de Ridder, D. , & de Visser, J. A. G. M. (2017). Genomics of adaptation depends on the rate of environmental change in experimental yeast populations. Molecular Biology and Evolution, 34, 2613–2626. http://academic.oup.com/mbe/article/34/10/2613/3896418/Genomics‐of‐Adaptation‐Depends‐on‐the‐Rate‐of, 10.1093/molbev/msx185 28957501

[yea3427-bib-0041] Greig, D. (2009). Reproductive isolation in *Saccharomyces* . Heredity, 102, 39–44. http://www.nature.com/articles/hdy200873, 10.1038/hdy.2008.73 18648383

[yea3427-bib-0042] Greig, D. , Travisano, M. , Louis, E. J. , & Borts, R. H. (2003). A role for the mismatch repair system during incipient speciation in *Saccharomyces* . Journal of Evolutionary Biology, 16, 429–437. 10.1046/j.1420-9101.2003.00546.x 14635842

[yea3427-bib-0043] Gresham, D. , Desai, M. M. , Tucker, C. M. , Jenq, H. T. , Pai, D. A. , Ward, A. , … Dunham, M. J. (2008). The repertoire and dynamics of evolutionary adaptations to controlled nutrient‐limited environments in yeast. PLoS Genetics, 4, e1000303 10.1371/journal.pgen.1000303 19079573 PMC2586090

[yea3427-bib-0044] Hamilton, J. A. , & Miller, J. M. (2016). Adaptive introgression as a resource for management and genetic conservation in a changing climate. Conservation Biology, 30, 33–41. 10.1111/cobi.12574 26096581

[yea3427-bib-0045] Hassold, T. , & Hunt, P. (2001). To err (meiotically) is human: The genesis of human aneuploidy. Nature Reviews Genetics, 2, 280–291. http://www.nature.com/articles/35066065, 10.1038/35066065 11283700

[yea3427-bib-0046] Hickman, M. A. , Paulson, C. , Dudley, A. , & Berman, J. (2015). Parasexual ploidy reduction drives population heterogeneity through random and transient aneuploidy in *Candida albicans* . Genetics, 200, 781–794. 10.1534/genetics.115.178020 25991822 PMC4512543

[yea3427-bib-0047] Hirakawa, M. P. , Chyou, D. E. , Huang, D. , Slan, A. R. , & Bennett, R. J. (2017). Parasex generates phenotypic diversity de novo and impacts drug resistance and virulence in *Candida albicans* . Genetics, 207, 1195–1211. 10.1534/genetics.117.300295 28912344 PMC5676243

[yea3427-bib-0048] Hong, J. , & Gresham, D. (2014). Molecular specificity, convergence and constraint shape adaptive evolution in nutrient‐poor environments. PLoS Genetics, 10, e1004041 10.1371/journal.pgen.1004041 24415948 PMC3886903

[yea3427-bib-0049] Hose, J. , Yong, C. M. , Sardi, M. , Wang, Z. , Newton, M. A. , & Gasch, A. P. (2015). Dosage compensation can buffer copy‐number variation in wild yeast. eLife, 4, 1–28. https://elifesciences.org/articles/05462 10.7554/eLife.05462 PMC4448642 25955966

[yea3427-bib-0050] Hou, J. , Friedrich, A. , de Montigny, J. , & Schacherer, J. (2014). Chromosomal rearrangements as a major mechanism in the onset of reproductive isolation in *Saccharomyces cerevisiae* . Current Biology, 24, 1153–1159. 10.1016/j.cub.2014.03.063 24814147 PMC4067053

[yea3427-bib-0051] Hu, G. , Liu, I. , Sham, A. , Stajich, J. E. , Dietrich, F. S. , & Kronstad, J. W. (2008). Comparative hybridization reveals extensive genome variation in the AIDS‐associated pathogen *Cryptococcus neoformans* . Genome Biology, 9, R41 10.1186/gb-2008-9-2-r41 18294377 PMC2374700

[yea3427-bib-0052] Hu, G. , Wang, J. , Choi, J. , Jung, W. H. , Liu, I. , Litvintseva, A. P. , … Kronstad, J. W. (2011). Variation in chromosome copy number influences the virulence of *Cryptococcus neoformans* and occurs in isolates from AIDS patients. BMC Genomics, 12, 526 10.1186/1471-2164-12-526 22032296 PMC3221739

[yea3427-bib-0053] Hunter, N. , Chambers, S. R. , Louis, E. J. , & Borts, R. H. (1996). The mismatch repair system contributes to meiotic sterility in an interspecific yeast hybrid. The EMBO Journal, 15, 1726–1733. http://www.ncbi.nlm.nih.gov/pubmed/8612597, 10.1002/j.1460-2075.1996.tb00518.x 8612597 PMC450085

[yea3427-bib-0054] Iantorno, S. A. , Durrant, C. , Khan, A. , Sanders, M. J. , Beverley, S. M. , Warren, W. C. , … Grigg, M. E. (2017). Gene expression in *Leishmania* is regulated predominantly by gene dosage. MBio, 8, e01393–e01317. 10.1128/mBio.01393-17 28900023 PMC5596349

[yea3427-bib-0055] Jaffe, M. , Sherlock, G. , & Levy, S. F. (2017). iSeq: A new double‐barcode method for detecting dynamic genetic interactions in yeast. G3: Genes|Genomes|Genetics, 7, 143–153. 10.1534/g3.116.034207 27821633 PMC5217104

[yea3427-bib-0056] Kao, K. C. , Schwartz, K. , & Sherlock, G. (2010). A genome‐wide analysis reveals no nuclear Dobzhansky‐Muller pairs of determinants of speciation between *S. cerevisiae* and *S. paradoxus*, but suggests more complex incompatibilities. PLoS Genetics, 6, e1001038 10.1371/journal.pgen.1001038 20686707 PMC2912382

[yea3427-bib-0057] Kassambara A. 2018 ggpubr: “ggplot2” based publication ready plots. https://cran.r‐project.org/package=ggpubr.

[yea3427-bib-0058] Kops, G. J. P. L. , Weaver, B. A. A. , & Cleveland, D. W. (2005). On the road to cancer: Aneuploidy and the mitotic checkpoint. Nature Reviews Cancer, 5, 773–785. 10.1038/nrc1714 16195750

[yea3427-bib-0059] Kovach, R. P. , Luikart, G. , Lowe, W. H. , Boyer, M. C. , & Muhlfeld, C. C. (2016). Risk and efficacy of human‐enabled interspecific hybridization for climate‐change adaptation: response to Hamilton and Miller (2016). Conservation Biology, 30, 428–430. 10.1111/cobi.12678 26918487

[yea3427-bib-0060] Kumar, P. , Lodge, R. , Raymond, F. , Ritt, J.‐F. , Jalaguier, P. , Corbeil, J. , … Tremblay, M. J. (2013). Gene expression modulation and the molecular mechanisms involved in Nelfinavir resistance in *Leishmania donovani* axenic amastigotes. Molecular Microbiology, 89, 565–582. 10.1111/mmi.12298 23782314

[yea3427-bib-0061] Kumaran, R. , Yang, S.‐Y. , & Leu, J.‐Y. (2013). Characterization of chromosome stability in diploid, polyploid and hybrid yeast cells. PLoS ONE, 8, e68094 10.1371/journal.pone.0068094 23874507 PMC3707968

[yea3427-bib-0062] Lauer, S. , Avecilla, G. , Spealman, P. , Sethia, G. , Brandt, N. , Levy, S. F. , & Gresham, D. (2018). Single‐cell copy number variant detection reveals the dynamics and diversity of adaptation. PLoS Biology, 16, e3000069 10.1371/journal.pbio.3000069 30562346 PMC6298651

[yea3427-bib-0063] Lengeler, K. B. , Cox, G. M. , & Heitman, J. (2001). Serotype AD strains of *Cryptococcus neoformans* are diploid or aneuploid and are heterozygous at the mating‐type locus. Infection and Immunity, 69, 115–122. 10.1128/IAI.69.1.115-122.2001 11119496 PMC97862

[yea3427-bib-0064] Lewis, M. D. , Llewellyn, M. S. , Gaunt, M. W. , Yeo, M. , Carrasco, H. J. , & Miles, M. A. (2009). Flow cytometric analysis and microsatellite genotyping reveal extensive DNA content variation in *Trypanosoma cruzi* populations and expose contrasts between natural and experimental hybrids. International Journal for Parasitology, 39, 1305–1317. 10.1016/j.ijpara.2009.04.001 19393242 PMC2731025

[yea3427-bib-0065] Li, W. , Averette, A. F. , Desnos‐Ollivier, M. , Ni, M. , Dromer, F. , & Heitman, J. (2012). Genetic diversity and genomic plasticity of *Cryptococcus neoformans* AD hybrid strains. G3: Genes|Genomes|Genetics, 2, 83–97. 10.1534/g3.111.001255 22384385 PMC3276195

[yea3427-bib-0066] Linder, R. A. , Greco, J. P. , Seidl, F. , Matsui, T. , & Ehrenreich, I. M. (2017). The stress‐inducible peroxidase TSA2 underlies a conditionally beneficial chromosomal duplication in *Saccharomyces cerevisiae* . G3: Genes|Genomes|Genetics, 7, 3177–3184. 10.1534/g3.117.300069 28743806 PMC5592942

[yea3427-bib-0067] Liti, G. , Barton, D. B. H. , & Louis, E. J. (2006). Sequence diversity, reproductive isolation and species concepts in *Saccharomyces* . Genetics, 174, 839–850. 10.1534/genetics.106.062166 16951060 PMC1602076

[yea3427-bib-0068] Liti, G. , Carter, D. M. , Moses, A. M. , Warringer, J. , Parts, L. , James, S. A. , … Louis, E. J. (2009). Population genomics of domestic and wild yeasts. Nature, 458, 337–341. 10.1038/nature07743 19212322 PMC2659681

[yea3427-bib-0069] Liu, H. , Krizek, J. , & Bretscher, A. (1992). Construction of a GAL1‐regulated yeast cDNA expression library and its application to the identification of genes whose overexpression causes lethality in yeast. Genetics, 132, 665–673. http://www.genetics.org/content/132/3/665 1468625 10.1093/genetics/132.3.665 PMC1205205

[yea3427-bib-0070] Lopandic, K. , Gangl, H. , Wallner, E. , Tscheik, G. , Leitner, G. , Querol, A. , … Tiefenbrunner, W. (2007). Genetically different wine yeasts isolated from Austrian vine‐growing regions influence wine aroma differently and contain putative hybrids between *Saccharomyces cerevisiae* and *Saccharomyces kudriavzevii* . FEMS Yeast Research, 7, 953–965. 10.1111/j.1567-1364.2007.00240.x 17484739

[yea3427-bib-0071] Lv, L. , Zhang, T. , Yi, Q. , Huang, Y. , Wang, Z. , Hou, H. , … Shi, Q. (2012). Tetraploid cells from cytokinesis failure induce aneuploidy and spontaneous transformation of mouse ovarian surface epithelial cells. Cell Cycle, 11, 2864–2875. 10.4161/cc.21196 22801546 PMC3419060

[yea3427-bib-0072] Macdonald, S. L. , Llewelyn, J. , Moritz, C. , & Phillips, B. L. (2017). Peripheral isolates as sources of adaptive diversity under climate change. Frontiers in Ecology and Evolution, 5, 1–10. 10.3389/fevo.2017.00088/full

[yea3427-bib-0073] Mancera, E. , Bourgon, R. , Brozzi, A. , Huber, W. , & Steinmetz, L. M. (2008). High‐resolution mapping of meiotic crossovers and non‐crossovers in yeast. Nature, 454, 479–485. http://www.nature.com/articles/nature07135, 10.1038/nature07135 18615017 PMC2780006

[yea3427-bib-0074] McFarlane, S. E. , & Pemberton, J. M. (2019). Detecting the true extent of introgression during anthropogenic hybridization. Trends in Ecology & Evolution, 34, 315–326. https://linkinghub.elsevier.com/retrieve/pii/S0169534718303057, 10.1016/j.tree.2018.12.013 30655011

[yea3427-bib-0075] Miller, J. M. , & Hamilton, J. A. (2016). Interspecies hybridization in the conservation toolbox: response to Kovach et al. (2016). Conservation Biology, 30, 431–433. 10.1111/cobi.12677 26918380

[yea3427-bib-0076] Millet, C. , Ausiannikava, D. , Le Bihan, T. , Granneman, S. , & Makovets, S. (2015). Cell populations can use aneuploidy to survive telomerase insufficiency. Nature Communications, 6, 8664 10.1038/ncomms9664 PMC4627575 26489519

[yea3427-bib-0077] Minning, T. A. , Weatherly, D. B. , Flibotte, S. , & Tarleton, R. L. (2011). Widespread, focal copy number variations (CNV) and whole chromosome aneuploidies in *Trypanosoma cruzi* strains revealed by array comparative genomic hybridization. BMC Genomics, 12, 139 10.1186/1471-2164-12-139 21385342 PMC3060142

[yea3427-bib-0078] Morard, M. , Macías, L. G. , Adam, A. C. , Lairón‐peris, M. , Pérez‐torrado, R. , Toft, C. , & Barrio, E. (2019). Aneuploidy and ethanol tolerance in *Saccharomyces cerevisiae* . Frontiers in Genetics, 10 10.3389/fgene.2019.00082 PMC6379819 30809248

[yea3427-bib-0079] Muhlfeld, C. C. , Kovach, R. P. , Jones, L. A. , Al‐Chokhachy, R. , Boyer, M. C. , Leary, R. F. , … Allendorf, F. W. (2014). Invasive hybridization in a threatened species is accelerated by climate change. Nature Climate Change, 4, 620–624. http://www.nature.com/articles/nclimate2252, 10.1038/nclimate2252

[yea3427-bib-0080] Mukherjee, A. , Boisvert, S. , do Monte‐Neto, R. L. , Coelho, A. C. , Raymond, F. , Mukhopadhyay, R. , … Ouellette, M. (2013). Telomeric gene deletion and intrachromosomal amplification in antimony‐resistant *Leishmania* . Molecular Microbiology, 88, 189–202. 10.1111/mmi.12178 23421749

[yea3427-bib-0081] Naumova, E. S. , Sadykova, A. Z. , Martynenko, N. N. , & Naumov, G. I. (2013). Molecular genetic characteristics of *Saccharomyces cerevisiae* distillers' yeasts. Microbiology, 82, 175–185. 10.1134/S0026261713020112 23808142

[yea3427-bib-0082] Ni, M. , Feretzaki, M. , Li, W. , Floyd‐Averette, A. , Mieczkowski, P. , Dietrich, F. S. , & Heitman, J. (2013). Unisexual and heterosexual meiotic reproduction generate aneuploidy and phenotypic diversity de novo in the yeast *Cryptococcus neoformans* . PLoS Biology, 11, e1001653 10.1371/journal.pbio.1001653 24058295 PMC3769227

[yea3427-bib-0083] Ochman, H. , Lawrence, J. G. , & Groisman, E. A. (2000). Lateral gene transfer and the nature of bacterial innovation. Nature, 405, 299–304. 10.1038/35012500 10830951

[yea3427-bib-0084] Pavelka, N. , Rancati, G. , Zhu, J. , Bradford, W. D. , Saraf, A. , Florens, L. , … Li, R. (2010). Aneuploidy confers quantitative proteome changes and phenotypic variation in budding yeast. Nature, 468, 321–325. 10.1038/nature09529 20962780 PMC2978756

[yea3427-bib-0085] Peris, D. , Langdon, Q. K. , Moriarty, R. V. , Sylvester, K. , Bontrager, M. , Charron, G. , … Hittinger, C. T. (2016). Complex ancestries of lager‐brewing hybrids were shaped by standing variation in the wild yeast *Saccharomyces eubayanus* . PLoS Genetics, 12, e1006155 10.1371/journal.pgen.1006155 27385107 PMC4934787

[yea3427-bib-0086] Peter, J. , de Chiara, M. , Friedrich, A. , Yue, J. X. , Pflieger, D. , Bergström, A. , … Schacherer, J. (2018). Genome evolution across 1,011 *Saccharomyces cerevisiae* isolates. Nature, 556, 339–344. http://www.nature.com/articles/s41586‐018‐0030‐5, 10.1038/s41586-018-0030-5 29643504 PMC6784862

[yea3427-bib-0087] Piotrowski, J. S. , Nagarajan, S. , Kroll, E. , Stanbery, A. , Chiotti, K. E. , Kruckeberg, A. L. , … Rosenzweig, F. (2012). Different selective pressures lead to different genomic outcomes as newly‐formed hybrid yeasts evolve. BMC Evolutionary Biology, 12, 46 http://www.biomedcentral.com/1471‐2148/12/46, 10.1186/1471-2148-12-46 22471618 PMC3372441

[yea3427-bib-0088] Poláková, S. , Blume, C. , Zárate, J. Á. , Mentel, M. , Jørck‐Ramberg, D. , Stenderup, J. , & Piškur, J. (2009). Formation of new chromosomes as a virulence mechanism in yeast *Candida glabrata* . Proceedings of the National Academy of Sciences, 106, 2688–2693. 10.1073/pnas.0809793106 PMC2637908 19204294

[yea3427-bib-0089] Prieto Barja, P. , Pescher, P. , Bussotti, G. , Dumetz, F. , Imamura, H. , Kedra, D. , … Späth, G. F. (2017). Haplotype selection as an adaptive mechanism in the protozoan pathogen *Leishmania donovani* . Nature Ecology & Evolution, 1, 1961–1969. 10.1038/s41559-017-0361-x 29109466

[yea3427-bib-0090] R Core Team . 2018 R: A language and environment for statistical computing. https://www.r‐project.org/.

[yea3427-bib-0091] Reis‐Cunha, J. L. , Rodrigues‐Luiz, G. F. , Valdivia, H. O. , Baptista, R. P. , Mendes, T. A. O. , de Morais, G. L. , … Bartholomeu, D. C. (2015). Chromosomal copy number variation reveals differential levels of genomic plasticity in distinct *Trypanosoma cruzi* strains. BMC Genomics, 16, 499 10.1186/s12864-015-1680-4 26141959 PMC4491234

[yea3427-bib-0092] Rhodes, J. , Desjardins, C. A. , Sykes, S. M. , Beale, M. A. , Vanhove, M. , Sakthikumar, S. , … Cuomo, C. A. (2017). Tracing genetic exchange and biogeography of *Cryptococcus neoformans* var. *grubii* at the global population level. Genetics, 207, 327–346. 10.1534/genetics.117.203836 28679543 PMC5586382

[yea3427-bib-0093] Ried, T. , Hu, Y. , Difilippantonio, M. J. , Ghadimi, B. M. , Grade, M. , & Camps, J. (2012). The consequences of chromosomal aneuploidy on the transcriptome of cancer cells. Biochimica et Biophysica Acta (BBA) ‐ Gene Regulatory Mechanisms, 1819, 784–793. https://linkinghub.elsevier.com/retrieve/pii/S1874939912000727, 10.1016/j.bbagrm.2012.02.020 22426433 PMC4737485

[yea3427-bib-0094] Rogers, D. W. , McConnell, E. , Ono, J. , & Greig, D. (2018). Spore‐autonomous fluorescent protein expression identifies meiotic chromosome mis‐segregation as the principal cause of hybrid sterility in yeast. PLoS Biology, 16, e2005066 10.1371/journal.pbio.2005066 30419022 PMC6258379

[yea3427-bib-0095] Rogers, M. B. , Hilley, J. D. , Dickens, N. J. , Wilkes, J. , Bates, P. A. , Depledge, D. P. , … Mottram, J. C. (2011). Chromosome and gene copy number variation allow major structural change between species and strains of *Leishmania* . Genome Research, 21, 2129–2142. 10.1101/gr.122945.111 22038252 PMC3227102

[yea3427-bib-0096] Romani, L. (2004). Immunity to fungal infections. Nature Reviews Immunology, 4, 11–24. http://www.nature.com/articles/nri1255, 10.1038/nri1255 14661066

[yea3427-bib-0097] Selmecki, A. , Forche, A. , & Berman, J. (2006). Aneuploidy and isochromosome formation in drug‐resistant *Candida albicans* . Science, 313, 367–370. 10.1126/science.1130164 16857942 PMC1717021

[yea3427-bib-0098] Selmecki, A. , Gerami‐Nejad, M. , Paulson, C. , Forche, A. , & Berman, J. (2008). An isochromosome confers drug resistance in vivo by amplification of two genes, ERG11 and TAC1. Molecular Microbiology, 68, 624–641. 10.1111/j.1365-2958.2008.06176.x 18363649

[yea3427-bib-0099] Selmecki, A. M. , Dulmage, K. , Cowen, L. E. , Anderson, J. B. , & Berman, J. (2009). Acquisition of aneuploidy provides increased fitness during the evolution of antifungal drug resistance. PLoS Genetics, 5, e1000705 10.1371/journal.pgen.1000705 19876375 PMC2760147

[yea3427-bib-0100] Selmecki, A. M. , Maruvka, Y. E. , Richmond, P. A. , Guillet, M. , Shoresh, N. , Sorenson, A. L. , … Pellman, D. (2015). Polyploidy can drive rapid adaptation in yeast. Nature, 519, 349–352. 10.1038/nature14187 25731168 PMC4497379

[yea3427-bib-0101] Shahid, M. , Han, S. , Yoell, H. , & Xu, J. (2008). Fitness distribution and transgressive segregation across 40 environments in a hybrid progeny population of the human‐pathogenic yeast *Cryptococcus neoformans* . Genome, 51, 272–281. 10.1139/G08-004 18356963

[yea3427-bib-0102] Sharp, N. P. , Sandell, L. , James, C. G. , & Otto, S. P. (2018). The genome‐wide rate and spectrum of spontaneous mutations differ between haploid and diploid yeast. Proceedings of the National Academy of Sciences, 115, E5046–E5055. 10.1073/pnas.1801040115 PMC5984525 29760081

[yea3427-bib-0103] Sheltzer, J. M. , & Amon, A. (2011). The aneuploidy paradox: Costs and benefits of an incorrect karyotype. Trends in Genetics, 27, 446–453. 10.1016/j.tig.2011.07.003 21872963 PMC3197822

[yea3427-bib-0104] Sheltzer, J. M. , Torres, E. M. , Dunham, M. J. , & Amon, A. (2012). Transcriptional consequences of aneuploidy. Proceedings of the National Academy of Sciences, 109, 12644–12649. 10.1073/pnas.1209227109 PMC3411958 22802626

[yea3427-bib-0105] Sirr, A. , Cromie, G. A. , Jeffery, E. W. , Gilbert, T. L. , Ludlow, C. L. , Scott, A. C. , & Dudley, A. M. (2015). Allelic variation, aneuploidy, and nongenetic mechanisms suppress a monogenic trait in yeast. Genetics, 199, 247–262. 10.1534/genetics.114.170563 25398792 PMC4286688

[yea3427-bib-0106] Smukowski Heil, C. S. , DeSevo, C. G. , Pai, D. A. , Tucker, C. M. , Hoang, M. L. , & Dunham, M. J. (2017). Loss of heterozygosity drives adaptation in hybrid yeast. Molecular Biology and Evolution, 34, 1596–1612. 10.1093/molbev/msx098 28369610 PMC5455960

[yea3427-bib-0107] Solieri, L. , Dakal, T. C. , & Bicciato, S. (2014). Quantitative phenotypic analysis of multistress response in *Zygosaccharomyces rouxii* complex. FEMS Yeast Research, 14, 586–600. 10.1111/1567-1364.12146 24533625

[yea3427-bib-0108] Stelkens, R. B. , Brockhurst, M. A. , Hurst, G. D. D. , & Greig, D. (2014). Hybridization facilitates evolutionary rescue. Evolutionary Applications, 7, 1209–1217. 10.1111/eva.12214 25558281 PMC4275092

[yea3427-bib-0109] Stelkens, R. B. , Brockhurst, M. A. , Hurst, G. D. D. , Miller, E. L. , & Greig, D. (2014). The effect of hybrid transgression on environmental tolerance in experimental yeast crosses. Journal of Evolutionary Biology, 7, 2507–2519. 10.1111/eva.12214 25262771

[yea3427-bib-0110] Sunshine, A. B. , Payen, C. , Ong, G. T. , Liachko, I. , Tan, K. M. , & Dunham, M. J. (2015). The fitness consequences of aneuploidy are driven by condition‐dependent gene effects. PLoS Biology, 13, e1002155 10.1371/journal.pbio.1002155 26011532 PMC4444335

[yea3427-bib-0111] Tan, Z. , Hays, M. , Cromie, G. A. , Jeffery, E. W. , Scott, A. C. , Ahyong, V. , … Dudley, A. M. (2013). Aneuploidy underlies a multicellular phenotypic switch. Proceedings of the National Academy of Sciences, 110, 12367–12372. 10.1073/pnas.1301047110 PMC3725063 23812752

[yea3427-bib-0112] Todd, R. T. , Forcher, A. , & Selmecki, A. M. (2017). Ploidy variation in fungi: Polyploidy, aneuploidy, and genome evolution. Microbiology Spectrum, 5, 1–21. 10.1128/microbiolspec.funk-0051-2016 PMC5656283 28752816

[yea3427-bib-0113] Torres, E. M. , Sokolsky, T. , Tucker, C. M. , Chan, L. Y. , Boselli, M. , Dunham, M. J. , & Amon, A. (2007). Effects of aneuploidy on cellular physiology and cell division in haploid yeast. Science, 317, 916–924. 10.1126/science.1142210 17702937

[yea3427-bib-0114] Ubeda, J.‐M. , Légaré, D. , Raymond, F. , Ouameur, A. , Boisvert, S. , Rigault, P. , … Ouellette, M. (2008). Modulation of gene expression in drug resistant *Leishmania* is associated with gene amplification, gene deletion and chromosome aneuploidy. Genome Biology, 9, R115 10.1186/gb-2008-9-7-r115 18638379 PMC2530873

[yea3427-bib-0115] Valdivia, H. O. , Almeida, L. V. , Roatt, B. M. , Reis‐Cunha, J. L. , Pereira, A. A. S. , Gontijo, C. , … Bartholomeu, D. C. (2017). Comparative genomics of canine‐isolated *Leishmania (Leishmania) amazonensis* from an endemic focus of visceral leishmaniasis in Governador Valadares, southeastern Brazil. Scientific Reports, 7, 40804 10.1038/srep40804 28091623 PMC5238499

[yea3427-bib-0116] Van de Peer, Y. , Mizrachi, E. , & Marchal, K. (2017). The evolutionary significance of polyploidy. Nature Reviews Genetics, 18, 411–424. 10.1038/nrg.2017.26 28502977

[yea3427-bib-0117] van den Broek, M. , Bolat, I. , Nijkamp, J. F. , Ramos, E. , Luttik, M. A. H. , Koopman, F. , … Daran, J. M. (2015). Chromosomal copy number variation in *Saccharomyces pastorianus* is evidence for extensive genome dynamics in industrial lager brewing strains. Applied and Environmental Microbiology, 81, 6253–6267. 10.1128/AEM.01263-15 26150454 PMC4542246

[yea3427-bib-0118] Voordeckers, K. , Kominek, J. , Das, A. , Espinosa‐Cantú, A. , de Maeyer, D. , Arslan, A. , … Verstrepen, K. J. (2015). Adaptation to high ethanol reveals complex evolutionary pathways. PLoS Genetics, 11, e1005635 10.1371/journal.pgen.1005635 26545090 PMC4636377

[yea3427-bib-0119] Weinstein, B. , & Solomon, F. (1990). Phenotypic consequences of tubulin overproduction in *Saccharomyces cerevisiae*: Differences between alpha‐tubulin and beta‐tubulin. Molecular and Cellular Biology, 10, 5295–5304. https://mcb.asm.org/content/10/10/5295, 10.1128/MCB.10.10.5295 2204812 PMC361218

[yea3427-bib-0120] Wickham, H. (2016). ggplot2: Elegant graphics for data analysis. New York: Springer‐Verlag http://ggplot2.org, 10.1007/978-3-319-24277-4

[yea3427-bib-0121] Yona, A. H. , Manor, Y. S. , Herbst, R. H. , Romano, G. H. , Mitchell, A. , Kupiec, M. , … Dahan, O. (2012). Chromosomal duplication is a transient evolutionary solution to stress. Proceedings of the National Academy of Sciences, 109, 21010–21015. 10.1073/pnas.1211150109 PMC3529009 23197825

[yea3427-bib-0122] Zhu, J. , Pavelka, N. , Bradford, W. D. , Rancati, G. , & Li, R. (2012). Karyotypic determinants of chromosome instability in aneuploid budding yeast. PLoS Genetics, 8, e1002719 10.1371/journal.pgen.1002719 22615582 PMC3355078

[yea3427-bib-0123] Zhu, Y. O. , Sherlock, G. , & Petrov, D. A. (2016). Whole genome analysis of 132 clinical *Saccharomyces cerevisiae* strains reveals extensive ploidy variation. G3: Genes|Genomes|Genetics, 6, 2421–2434. 10.1534/g3.116.029397 27317778 PMC4978896

