# Supplementary figures and images for: Aneuploidy in yeast: Segregation error or adaptation mechanism?
Source: Yeast. 2019 Aug 1;36(9):525–39. doi: 10.1002/yea.3427 (PMC6772139; doi:10.1002/yea.3427)

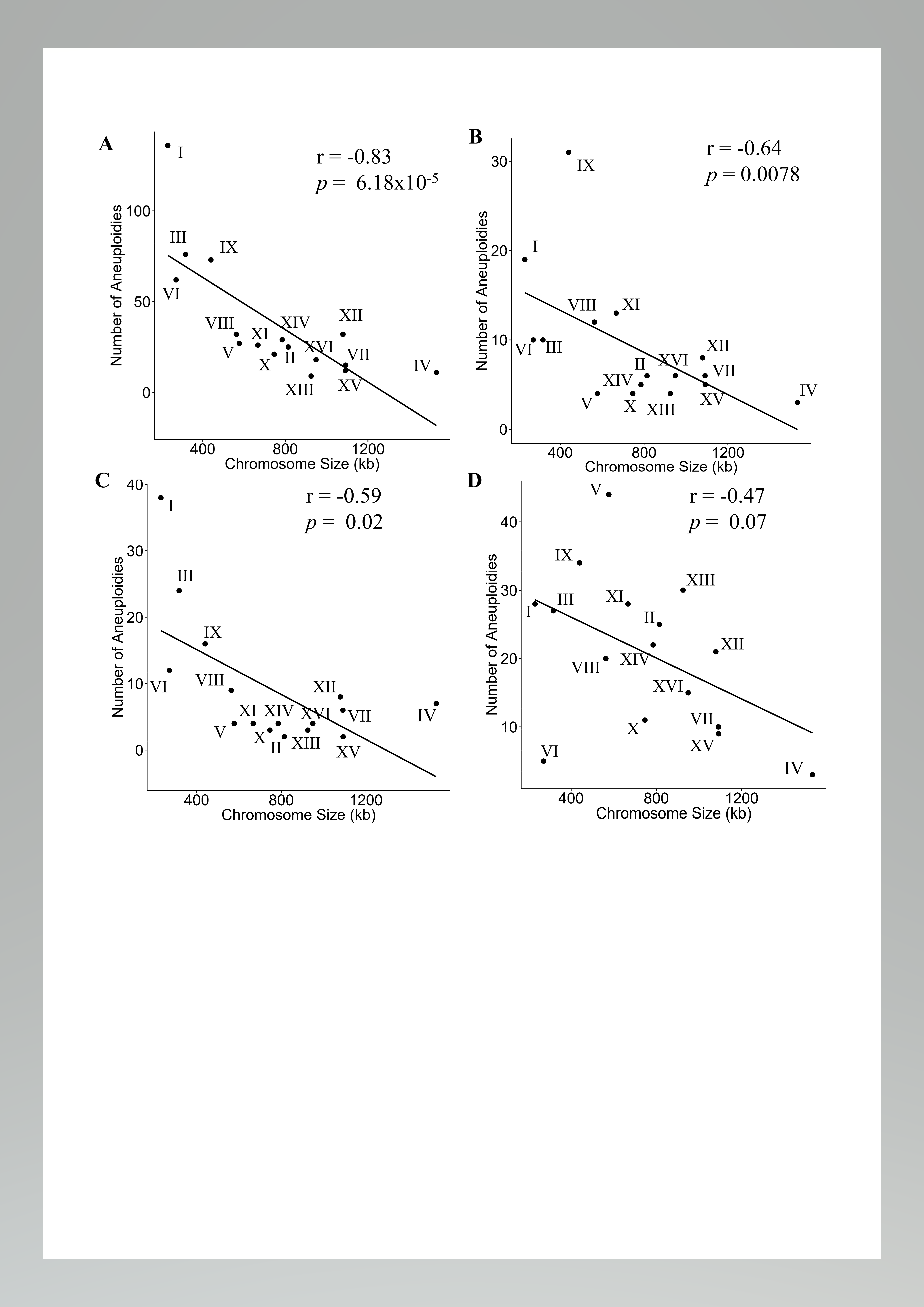

Supplement: Supplementary file 4 — Figure S1. Scatter plots of the data from Supplementary Tables 2. This includes industrial strains (A), pathogenic strains (B), wild strains (C) and experimental strains (D). Regression line indicates significant correlation. Analyses and graphs were made using R version 3.5.1 (Feather Spray; R Core Team, 2018) with the packages ggplot2 (Wickham, 2016), ggpubr (Kassambara, 2018) and magrittr (Bache & Wickham, 2014). [file YEA-36-525-s004.tiff]
